# Supplementary figures and images for: Effects of coconut water on blood sugar and retina of rats with diabetes
Source: PeerJ. 2021 Jan 29;9:e10667. doi: 10.7717/peerj.10667 (PMC7849505; doi:10.7717/peerj.10667)

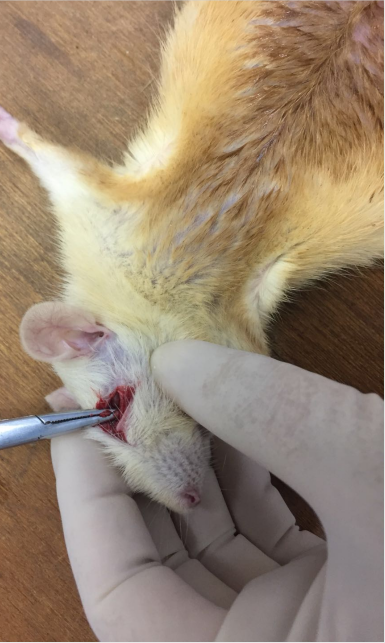

Supplement: Supplemental Information 1 [file peerj-09-10667-s001.zip › supporting information for experimental manipulation/fig1s.png]

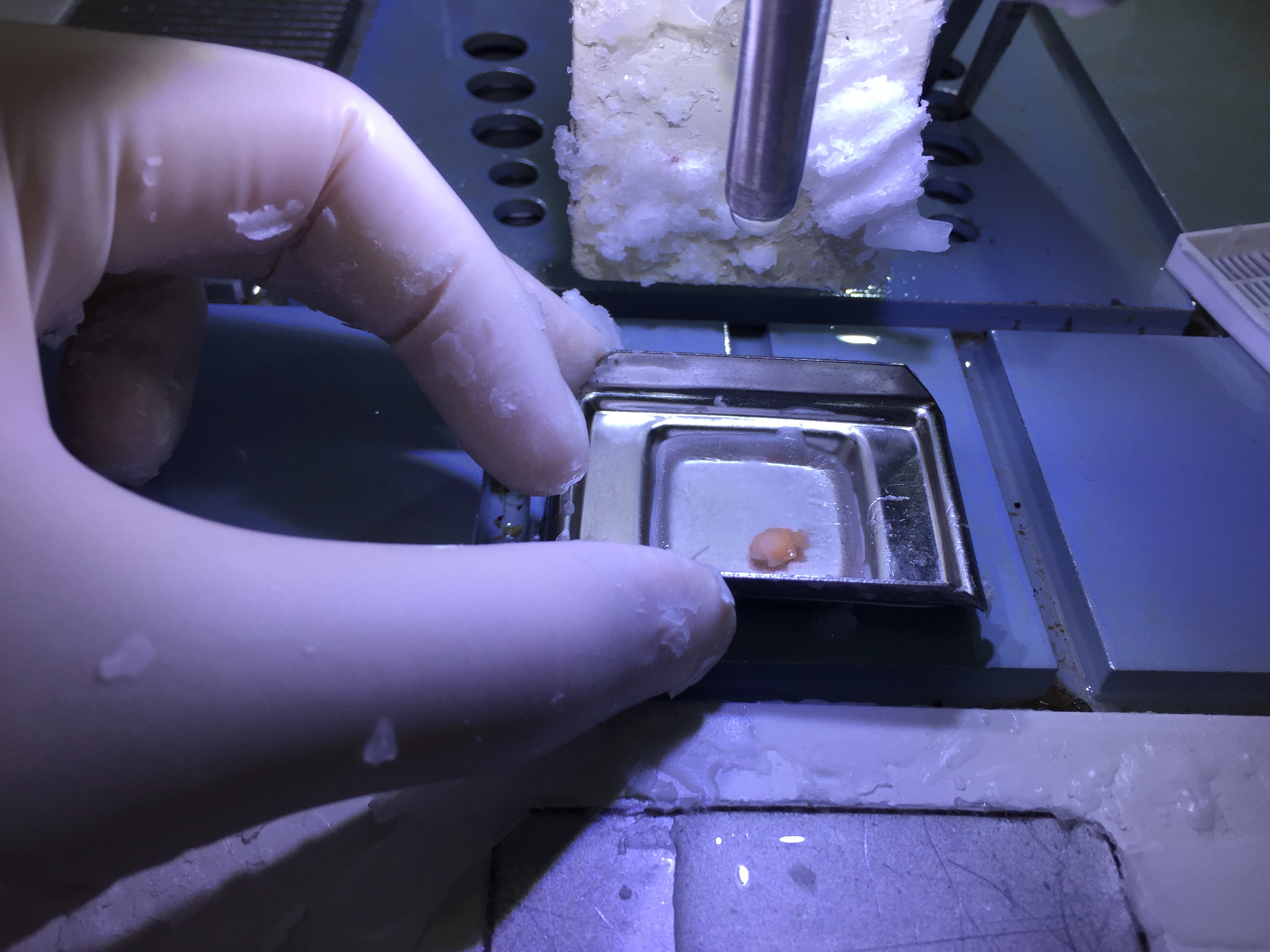

Supplement: Supplemental Information 1 [file peerj-09-10667-s001.zip › supporting information for experimental manipulation/fig2s.jpg]

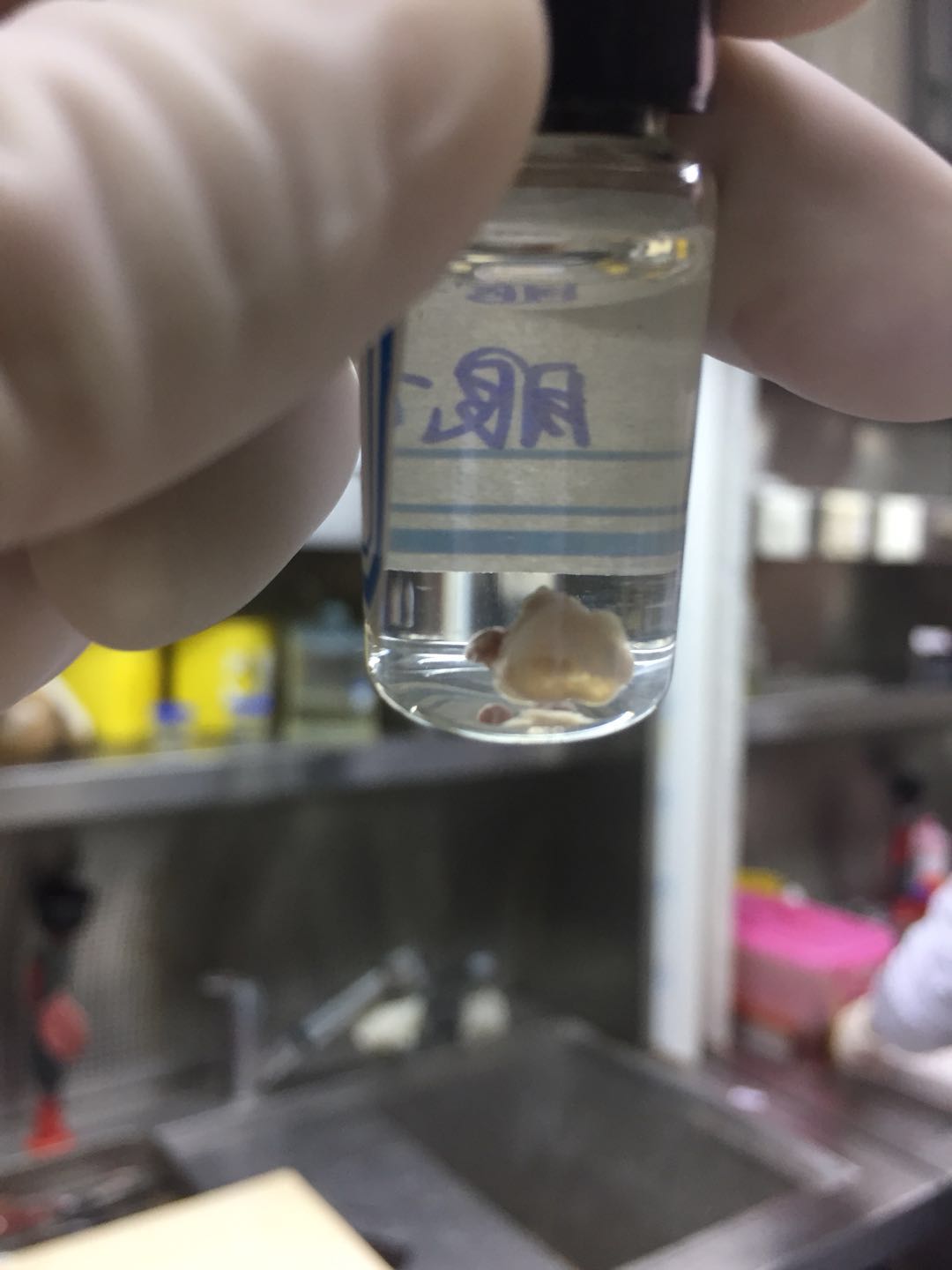

Supplement: Supplemental Information 1 [file peerj-09-10667-s001.zip › supporting information for experimental manipulation/fig3s.jpg]

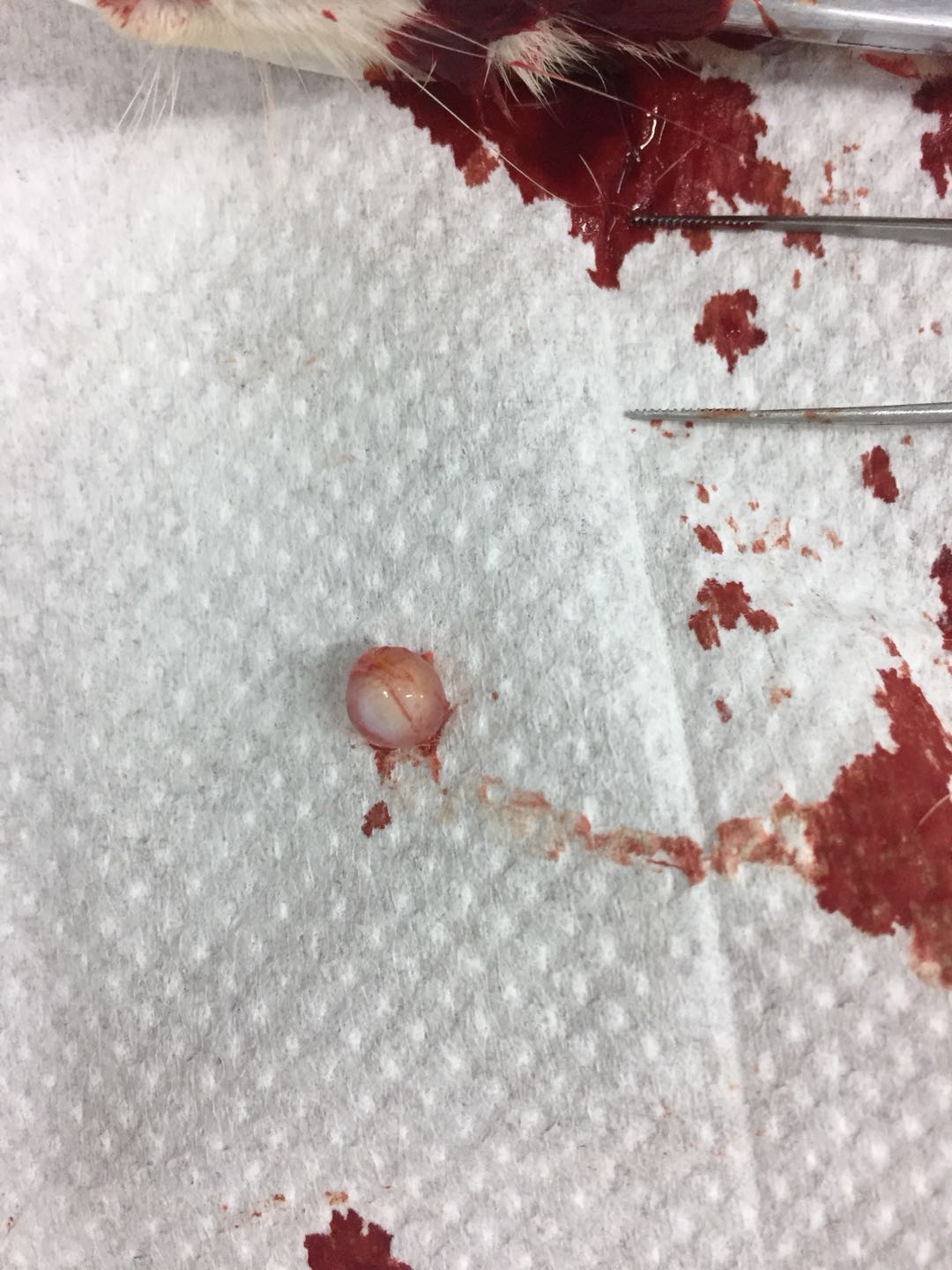

Supplement: Supplemental Information 1 [file peerj-09-10667-s001.zip › supporting information for experimental manipulation/fig4s.jpg]

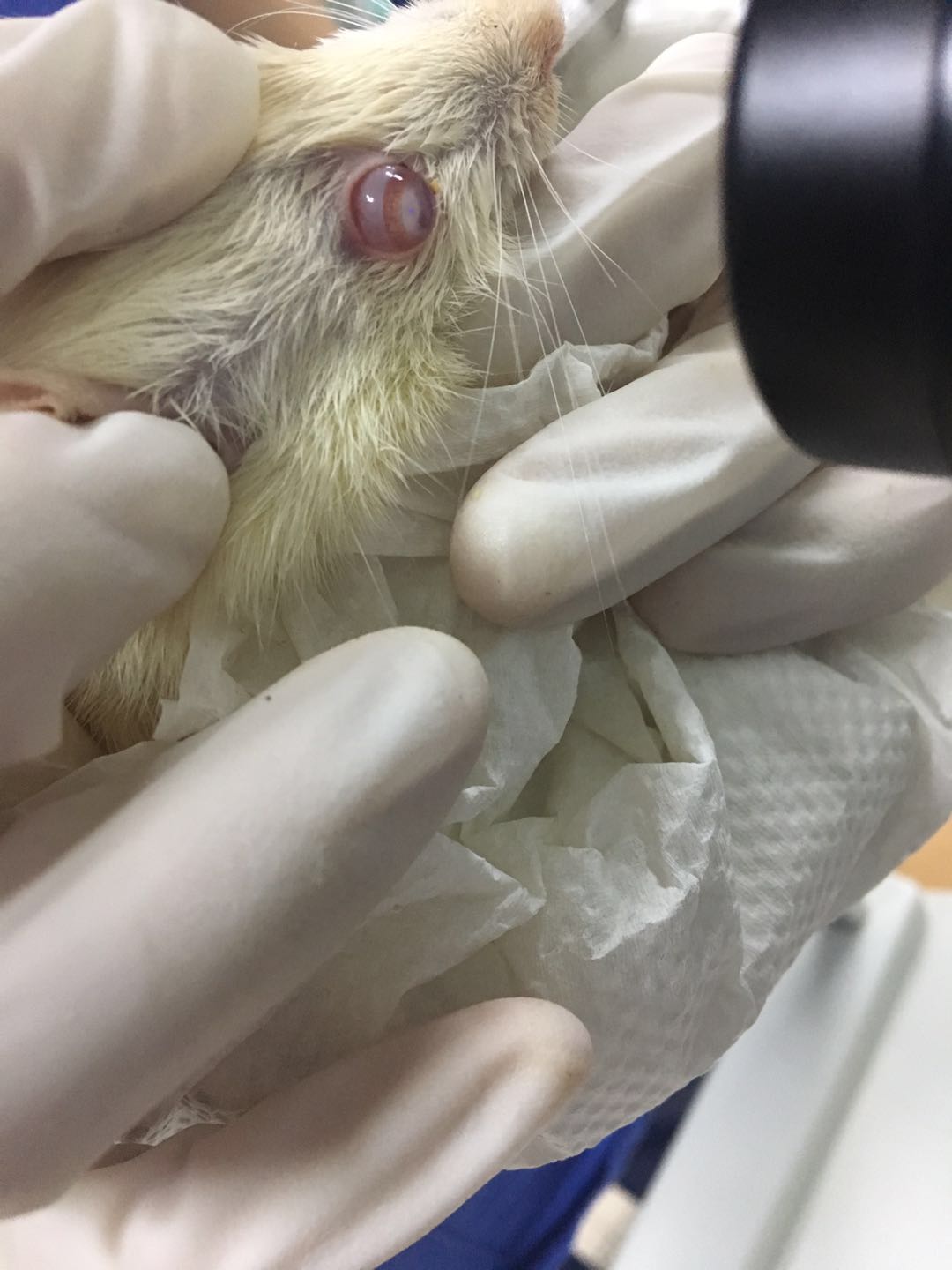

Supplement: Supplemental Information 1 [file peerj-09-10667-s001.zip › supporting information for experimental manipulation/fig5s.jpg]

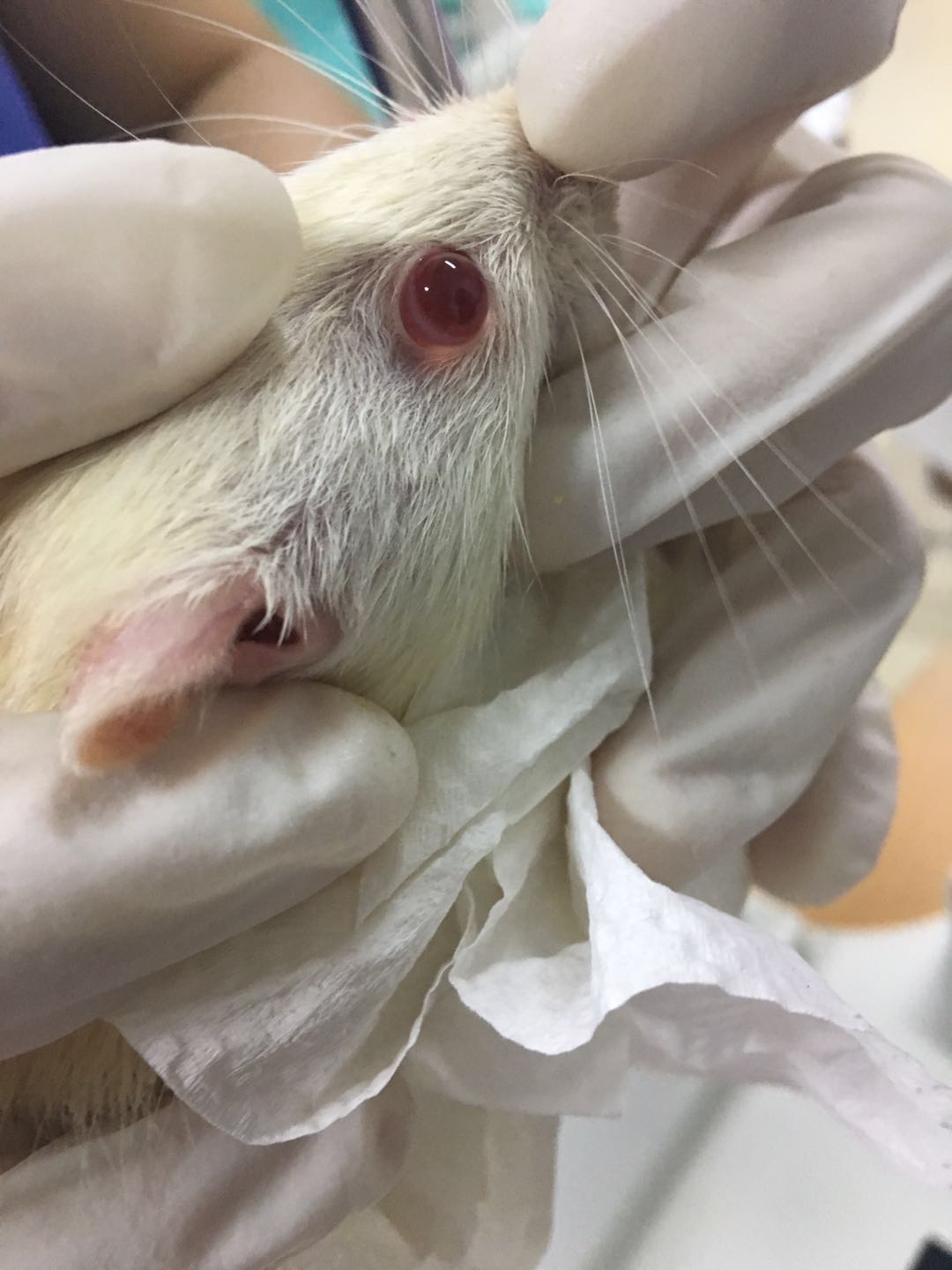

Supplement: Supplemental Information 1 [file peerj-09-10667-s001.zip › supporting information for experimental manipulation/fig6s.jpg]

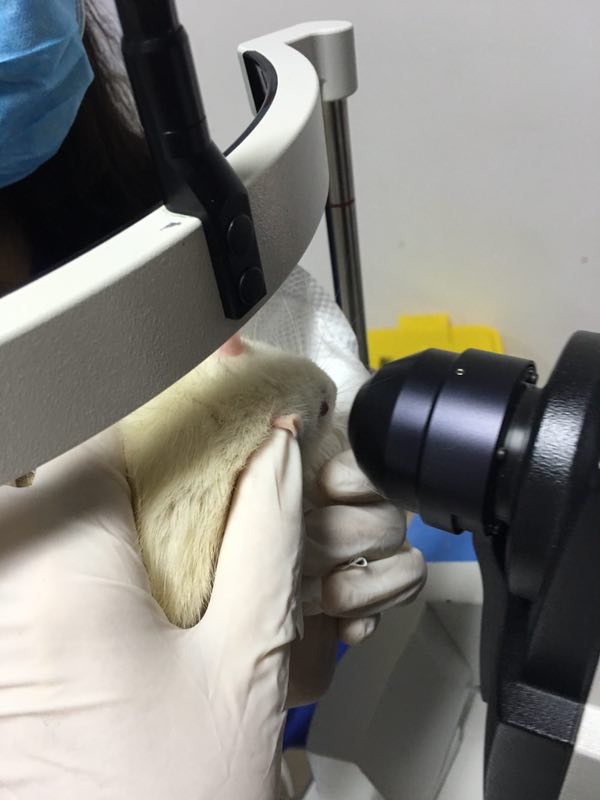

Supplement: Supplemental Information 1 [file peerj-09-10667-s001.zip › supporting information for experimental manipulation/fig7s.jpg]

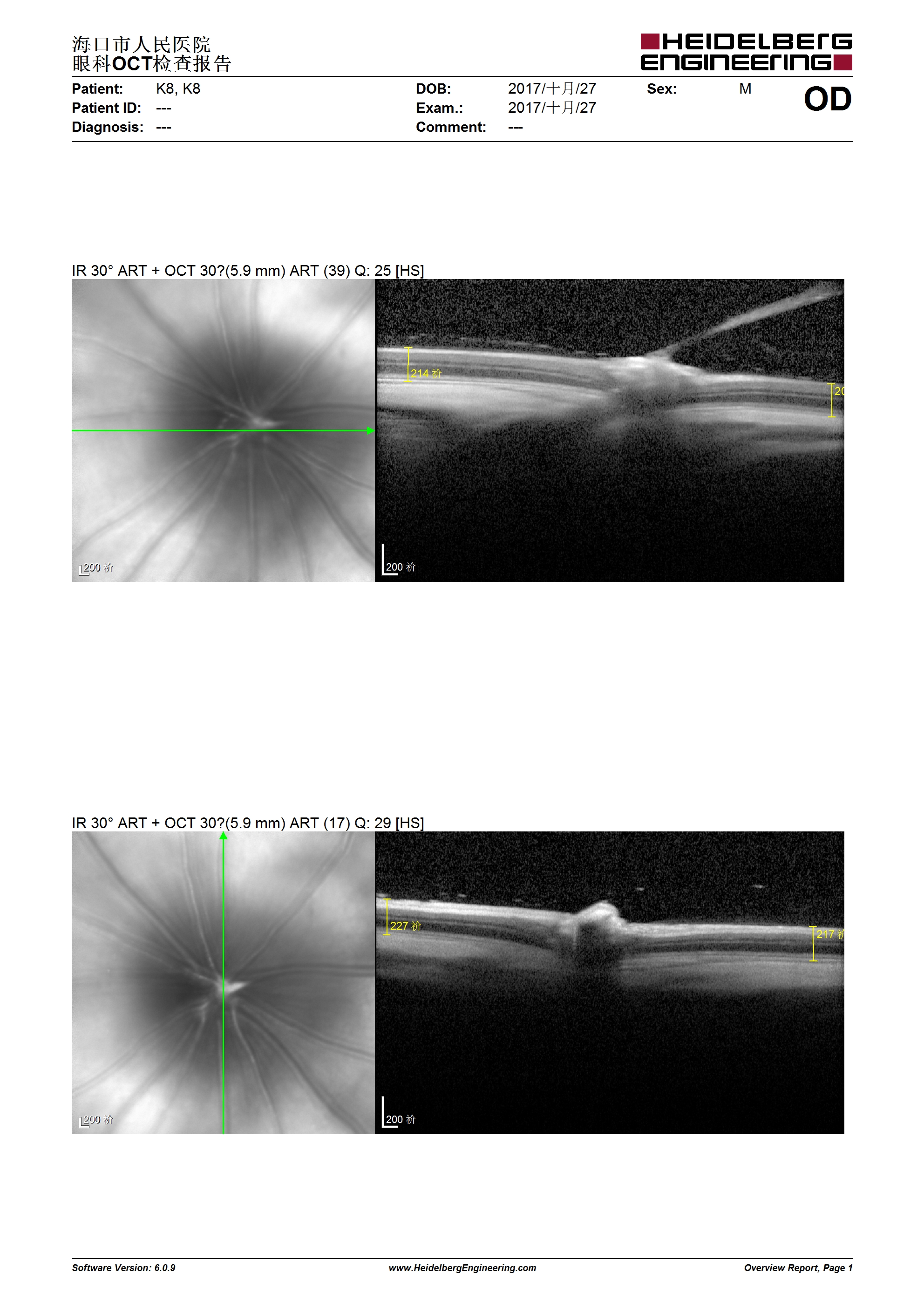

Supplement: Supplemental Information 1 [file peerj-09-10667-s001.zip › supporting information for experimental manipulation/fig8s.JPG]

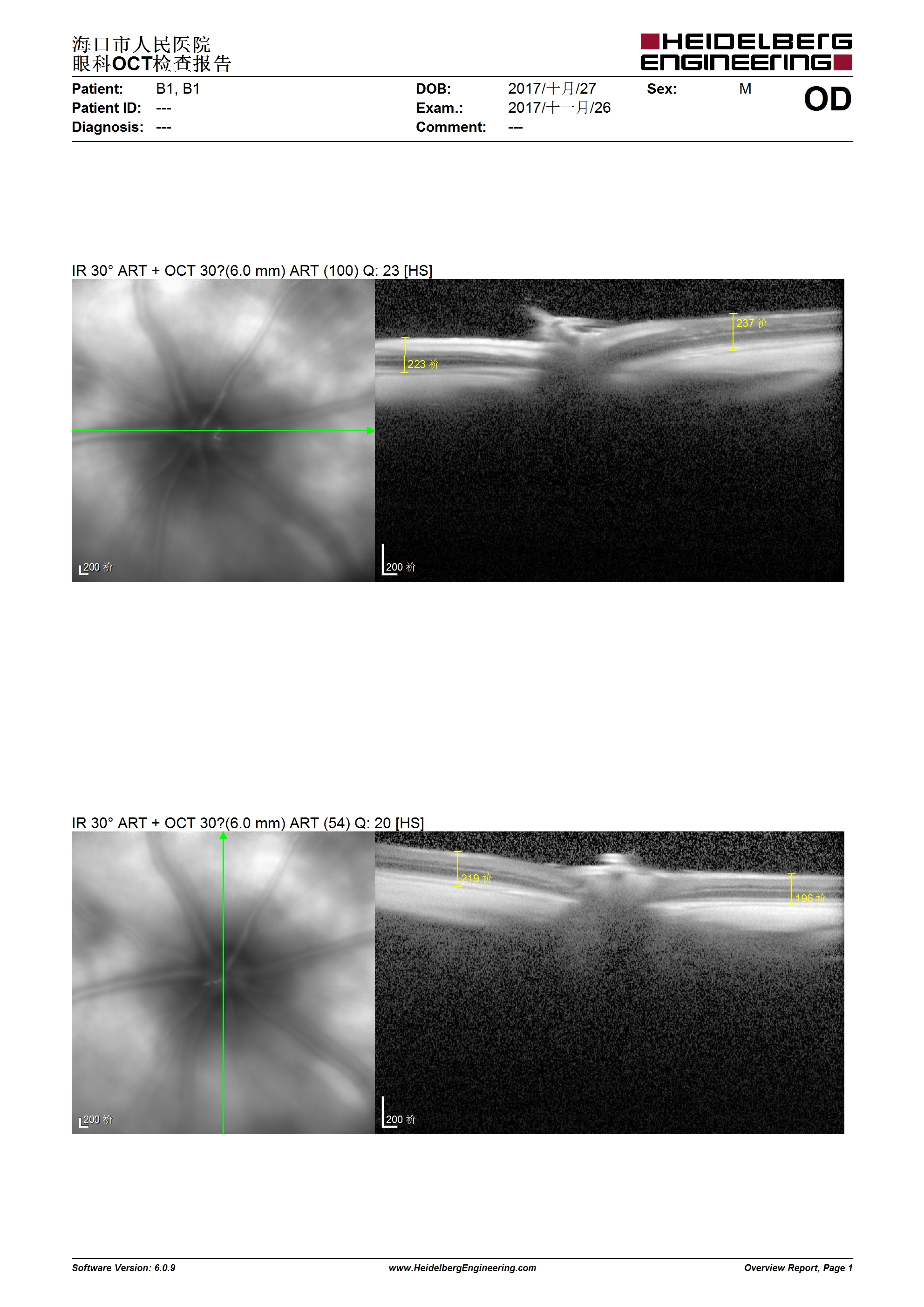

Supplement: Supplemental Information 1 [file peerj-09-10667-s001.zip › supporting information for experimental manipulation/fig9s.JPG]
